# A family of small cyclic amphipathic peptides (SCAmpPs) genes in citrus

**DOI:** 10.1186/s12864-015-1486-4

**Published:** 2015-04-16

**Authors:** William R Belknap, Kent F McCue, Leslie A Harden, William H Vensel, Michael G Bausher, Ed Stover

**Affiliations:** USDA-ARS, Western Regional Research Center, Albany, CA USA; USDA-ARS, U. S. Horticultural Research Laboratory, Fort Pierce, FL USA

**Keywords:** *Citrus sinensis*, *Citrus clementina*, *Poncirus trifoliata*, Ribosomally synthesized

## Abstract

**Background:**

Citrus represents a crop of global importance both in economic impact and significance to nutrition. Citrus production worldwide is threatened by the disease Huanglongbing (HLB), caused by the phloem-limited pathogen *Candidatus* Liberibacter spp.. As a source of stable HLB-resistance has yet to be identified, there is considerable interest in characterization of novel disease-associated citrus genes.

**Results:**

A gene family of Small Cyclic Amphipathic Peptides (SCAmpPs) in citrus is described. The citrus genomes contain 100–150 SCAmpPs genes, approximately 50 of which are represented in the citrus EST database. These genes encode small ~50 residue precursor proteins that are post-translationally processed, releasing 5–10 residue cyclic peptides. The structures of the SCAmpPs genes are highly conserved, with the small coding domains interrupted by a single intron and relatively extended untranslated regions. Some family members are very highly transcribed in specific citrus tissues, as determined by representation in tissue-specific cDNA libraries. Comparison of the ESTs of related SCAmpPs revealed an unexpected evolutionary profile, consistent with targeted mutagenesis of the predicted cyclic peptide domain. The SCAmpPs genes are displayed in clusters on the citrus chromosomes, with apparent association with receptor leucine-rich repeat protein arrays. This study focused on three SCAmpPs family members with high constitutive expression in citrus phloem. Unexpectedly high sequence conservation was observed in the promoter region of two phloem-expressed SCAmpPs that encode very distinct predicted cyclic products. The processed cyclic product of one of these phloem SCAmpPs was characterized by LC-MS-MS analysis of phloem tissue, revealing properties consistent with a K^+^ ionophore.

**Conclusions:**

The SCAmpPs amino acid composition, protein structure, expression patterns, evolutionary profile and chromosomal distribution are consistent with designation as ribosomally synthesized defense-related peptides.

**Electronic supplementary material:**

The online version of this article (doi:10.1186/s12864-015-1486-4) contains supplementary material, which is available to authorized users.

## Background

Plants produce a broad array of peptides, short proteins of approximately 50 residues or less, largely involved in defense processes [1]. With the exception of phytochelatins [2], these plant peptides are ribosomally synthesized either as mature products or precursors that are post-translationally modified [3]. The plant peptides vary in size, composition, and mode of action, and many have been associated with defense against pathogens and predators.

These defense-related peptides accumulate either constitutively or are induced by injury/infection [4]. The plant defensins, for example, are encoded by a multigene family with small (45–54 residue) cysteine-rich protein products. While defensins can constitutively accumulate in plant tissues, specific family members are induced by a variety of biotic and abiotic stresses [4]. Defensin gene products have been shown to have anti-microbial activities, and have been successfully employed as transgene products in number of plants, including citrus [5].

In a study designed to identify citrus genes transcriptionally up-regulated by insect herbivory, a short transcript encoding an unknown 50-residue protein was identified [6]. Designated CsV03-3, in citrus leaves this transcript was shown to accumulate in response to application of methyl jasmonate, salicylic acid, abscisic acid and abiotic stress [7]. Three additional transcripts with >90% identity to CsV03-3 were identified in the EST database, indicating that it was a member of a gene family. The small proteins encoded by the transcripts were shown to bind dsDNA, and the family was designated as a new class of defense-related nucleic acid binding proteins [7]. However, unrelated investigations of citrus peptides with potential pharmaceutical applications [8,9] identified small cyclic citrus peptides with precursors closely related to the CsV03 family, suggesting CsV03 membership in a more diverse gene family encoding cyclic peptides [10].

Here we describe the structure, expression, evolutionary profile and genomic distribution of members of a diverse gene family of CsV03-related SCAmpPs. In addition, we show that one of the members, highly expressed in phloem tissue, encodes a cyclic peptide with the potential to act as a K^+^ carrier ionophore.

## Results

### SCAmpPs Peptides

**Figure 1:**
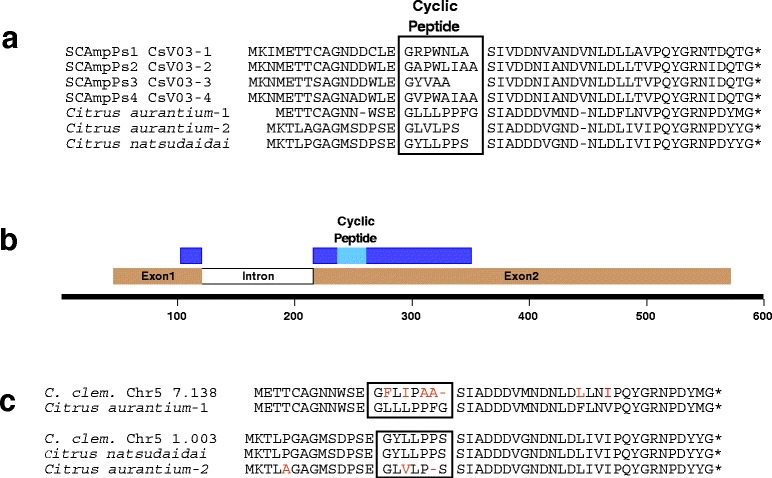
Alignment of SCAmpPs precursor proteins and structure of SCAmpPs genes. **(a)** Alignment of the precursor deduced amino acid sequences SCAmpPs-1 (NCBI accession EF175924), SCAmpPs-2 (EF175926), SCAmpPs-3 (EF175925), SCAmpPs-4 (EF175927), *Citrus aurantium*-1 (EY850721), *Citrus aurantium*-2 (EY848546) and *Citrus natsudaidai* (BB999724). Boxes indicate predicted cyclic peptide product domains. **(b)** Representation of a model citrus SCAmpPs gene. Locations of precursor codons are indicated in dark blue, cyclic peptide product codons in light blue. Exon and intron positions indicated by brown and white boxes. **(c)** Deduced amino acid sequences of *C. clementina* SCAmpPs located on chromosome 5 at positions 7.138 (NCBI accession FC928056) and 1.003 Mb (FC872825).

While the four previously described CsV03 transcripts were identified as encoding nucleic acid binding proteins [7], these proteins show considerable similarity to three cyclic-peptide precursors characterized in unrelated studies (Figure 1a) [8-10]. The two cyclic peptides from *C. aurantium* as well as the one from *C. natsudaidai* are in all likelihood derived from precursor proteins closely related to the four CsV03 proteins (Figure 1c) [10]. This implies that the CsV03 genes belong to a larger, and more diverse, family than previously indicated [7]. It has recently been suggested that small peptides cyclized N-to-C and lacking disulfides be referred to as orbitides [3].

**Figure 2:**
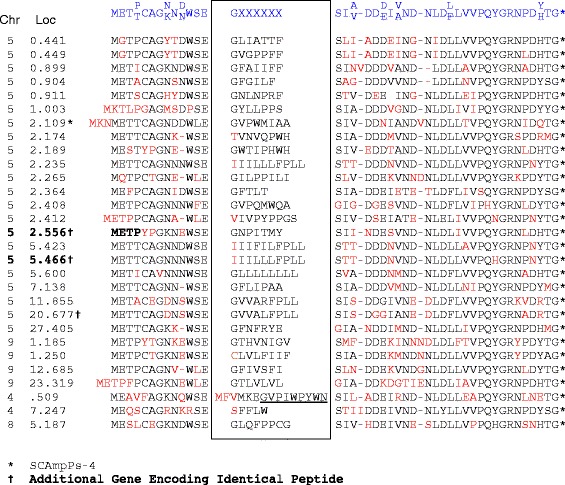
Alignment of SCAmpPs precursor proteins. Deduced SCAmpPs precursor protein sequences from citrus for gene products found in both the *Citrus sinensis* and *Citrus clementina* genomes as well as the citrus EST database. The predicted cyclic peptide product sequences are indicated, as well as the chromosome number and position (Mb) on the encoding gene *Citrus clementina* genome. The SCAmpPs precursor consensus sequence is indicated in blue.

In order to more fully characterize this gene family, a consensus sequence (Figure 2) was used to probe the *C. sinensis* [11] and *C. clementina* [12] genome assemblies. A total of 102 loci with similarity to the consensus sequence were identified on the *C. clementina* genome (Additional file 1), and of these 36 were associated with ESTs (≥99% identity) indicating that they represent expressed genes. A total of 150 similar loci were identified on the *C. sinensis* draft genome, 46 of which are associated with ESTs. The expressed genes had the structure indicated on Figure 1b, with two exons separated by an approximately 100 bp intron. While the coding sequences of these genes (approximately150 bp) are short, the transcripts contain relatively long 5’ and 3′ UTRs resulting in transcripts of approximately 450 bp (Figure 1b). Thirty-three of the expressed genes in *C. clementina* have at least 99% identity to the *C. sinensis* orthologs. The deduced amino acid sequences of the encoded precursor proteins, verified in two independent assemblies and the EST database, are aligned in Figure 2.

The alignments shown in Figure 1c support identification of the proteins in Figure 2 as precursors processed to the small predicted cyclic final products indicated. All three of the previously verified citrus precursors [8,9] align with specific expressed *C. clementina* deduced proteins. As a majority of the deduced cyclic products contain a combination of both non-polar/hydrophobic, and polar/charged residues, members of this gene family are referred to as Small Cyclic Amphipathic Peptides, or SCAmpPs. The previously described cyclic proteins from *Citrus aurantium* and *Citrus natsudaidai* are clearly members of this set (Figure 1c).

The SCAmpPs precursor product from 0.509 Mb on *C. clementina* chromosome 4 (Figure 2) has been entered to align the -WSE- amino-terminal processing site proposed for the other precursors. However, the structural features of the deduced precursor suggests that processing of this peptide results in the underlined GVPIWPYWN predicted cyclic peptide.

The processing of the Caryophyllaceae orbitide precursors in developing seeds to final, cyclic, products has been shown to occur in two steps [10,13]. Initially, the precursors are cleaved at the N-terminal end of the cyclic product by an oligopeptidase, followed by secondary cleavage at the cyclic peptide C-terminus and formation of the final product via a specific cyclase [13]. While there is no sequence similarity between the citrus and Caryophyllaceae precursors, their structural similarity [10] suggests a similar biosynthetic pathway.

The amino acid content of the SCAmpPs predicted cyclic peptides are enriched in non-polar amino acids (tryptophan, phenylalanine, proline, leucine, isoleucine) relative to expected frequencies [14], and while there is an overall decrease in charged residues, the ratio of basic to acidic amino acids is high in the SCAmpPs peptides (Additional file 2). This composition is consistent with peptide attachment and insertion into biological membranes [15].

### SCAmpPs transcription

Based on representation in the available citrus EST database [16], the individual SCAmpPs gene family members can have strikingly different transcriptional profiles (Additional file 3). For example representation of individual SCAmpPs in cDNA libraries from *C. sinensis* phloem (NCBI LIBEST_017673, Bausher, M., Shatters, R., Dang, P., Hunter, W. and Niedz, R., unpublished, 2005) and *C. clementina* ovary abscission zone C (NCBI LIBEST_019157 [17]) can be compared. SCAmpPs-2, SCAmpPs-3 and SCAmpPs-4 are highly represented in the phloem and under-represented in the ovary abscission zone, while the reverse is true of SCAmpPs-5 and SCAmpPs-6 (Additional file 3).

**Figure 3:**
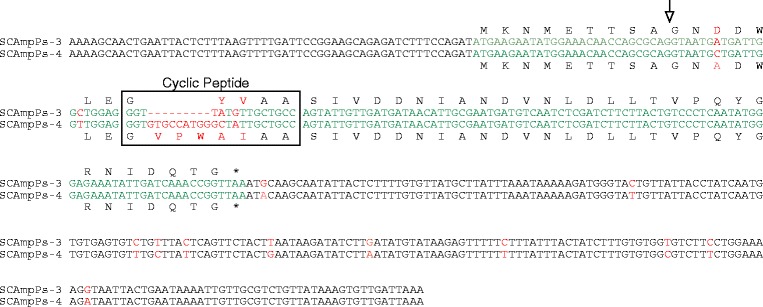
Alignment of SCAmpPs-3 and SCAmpPs-4 mRNA [7]. SCAmpPs3 (NCBI accession EF175925) and SCAmpPs4 (EF175927). Coding domains are indicated in green, mismatches in red. The position of the intron relative to the genomic sequence is indicated by the arrow.

A sequence comparison of ESTs encoding SCAmpPs-3 and SCAmpPs-4 is shown in Figure 3. These ESTs are identical prior to the intron, and diverge thereafter. While these mRNAs share 97% identity outside the predicted cyclic peptide domains, the cyclic peptide coding sequences more divergent and result in five residue SCAmpPs-3 (GYVAA) and eight residue SCAmpPs-4 (GVPWAIAA) cyclic peptide products that share only three common residues. A hypervariable domain is clearly apparent in the alignment as the domain encoding the final cyclic product. These two mRNAs share 100% identity in the domain 5′ to the intron processing locus with less conservation 3′ to the cyclic product.

**Figure 4:**
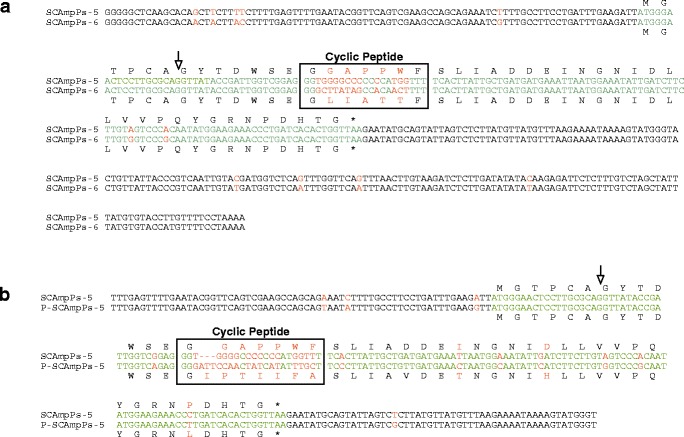
Alignment of citrus SCAmpPs-5 with SCAmpPs-6 and ortholog from Poncirus trifoliata mRNA. **(a)** Alignment of SCAmpPs-5 and SCAmpPs-6 ESTs. SCAmpPs-5 (NCBI accession FC872925) and SCAmpPs-6 (FC872592). Coding domains are indicated in green, mismatches in red. Position of the intron indicated as in Figure 3. **(b)** Alignment of SCAmpPs-5 EST and an orthologous EST P-SCAmpPs-5 (NCBI Accession FE896141) annotated as in **(a)** above.

The pattern of sequence divergence, specifically targeting the predicted cyclic peptide-coding domain, is observed in other SCAmpPs ESTs with similar expression profiles (based on representation in the database). For example, an alignment of SCAmpPs-5 and SCAmpPs-6 ESTs is shown in Figure 4a. While these ESTs share 97% identity outside the cyclic peptide domain (95% overall identity), the cyclic peptide domains share only 43% identity resulting in cyclic peptides (SCAmpPs-5 GGAPPWF, SCAmpPs-6 GLIATTF) that share only two common residues (Figure 4a). This pattern is also observed in comparison of the SCAmpPs-5 EST with an orthologous EST from *P. trifoliata*, an evolutionary divergence of approximately 4 million years [18]. These transcripts share 95% identity outside the cyclic peptide domain, while the localized changes in the cyclic peptide domain result in 7 and 8 residue peptides that share a single common residue (Figure 4b).

The pattern of hypervariability within the predicted cyclic peptide-coding region is a common feature within paralogous/orthologous collections of SCAmpPs-encoding ESTs (Additional file 4). This type of hypervariability is common in gene families that mediate interactions between organisms, the conotoxins (snail toxins) providing perhaps the best example [19]. The accelerated mutation rates associated with the cyclic peptide domains could reflect either a strong positive selection or specific hypermutation. In the conotoxin case, a targeted mutagenic process producing short hypervariable stretches within the mature toxin domain has been proposed [20]. Alternative mechanisms for targeted changes within the conotoxin coding region based on formation of highly stable secondary structures within conserved regions [21] do not appear applicable in the SCAmpPs genes as they lack obvious potential for such secondary structures in the conserved regions.

### Genomic structure of the citrus phloem SCAmpPs-3 and SCAmpPs-4 genes

While coding for distinct cyclic peptide products, the first exons of SCAmpPs-3 and SCAmpPs-4 mRNAs are identical (Figure 3). The *C. sinensis* Chr3 assembly [11] contains single SCAmpPs-3 (3.555 Mb, contains two tandem transcribed regions separated by 200 bp) and SCAmpPs-4 (3.635 Mb) loci. The SCAmpPs-3 and SCAmpPs-4 genes in this assembly have similar 5′ domains, with a 1.9 kb relative deletion 200 bp 5′ to the SCAmpPs-3 transcription start. The *C. clementina* Chr5 assembly [12] does not contain a SCAmpPs-3 gene, but has a SCAmpPs-4 gene at 2.109 Mb with a 5′ domain similar to the *C. sinensis* SCAmpPs-4 gene. The *C. clementina* Chr5 assembly also contains a SCAmpPs-4 –related pseudogene at 2.118 Mb. To evaluate the extent of conservation between these two genes, PCR primers were designed corresponding to 5′ and 3′ sequences conserved on both SCAmpPs-4 genes as well as *C. sinensis* SCAmpPs-3. These primers were used to amplify the genes from *C. clementina* genomic DNA.

**Figure 5:**
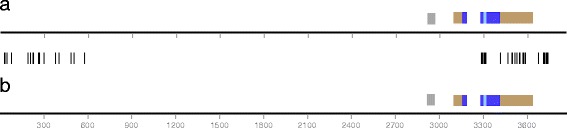
PCR Amplification of SCAmpPs-3 and SCAmpPs-4 genes. **(a)** gSCAmpPs-3-1, **(b)** gSCAmpPs-4-2. Exon, coding and cyclic peptide regions are indicated as in Figure 1. The grey boxes indicate the variable domains (Additional file 5). The black lines between the two sequences indicate the positions of single base pair differences.

The primers were expected to amplify products with 3.1 kb of 5′ and 250 bp of 3′ sequence in relation to the SCAmpPs-4 gene on the *C. sinensis* Chr3 assembly. The sequences of two SCAmps-3 and three SCAmpPs-4 genes amplified from *C. clementina* were determined, as well as a SCAmpPs-3 gene amplified from the rootstock Carrizo. The source of the 5′ identity of the SCAmpPs-3 and SCAmpPs-4 ESTs (Figure 3) is shown in Figure 5, in which the sequences of a SCAmpPs-3 and SCAmpPs-4 genomic PCR products are compared. The two PCR products (gSCAmpPs-3-1 and gSCAmpPs-4-2) with a 5′ promoter element of 3109 bp share identical sequence from a position 6 bp 3′ to the intron (Figure 3) to a position −2.5 kb relative to the transcription start. These two genes, which produce very different predicted cyclic products (Figure 1), therefore share the same 2.5 kb proximal promoter sequence, as well as first exon and intron sequences. The two additional SCAmpPs-4 PCR products had a 5′ sequence of 3128 bp due to a short variable region (46 or 65 bp) 130 bp 5′ to the transcription start (Figure 5, Additional file 5). Upstream of this variable domain the promoter sequences for the SCAmpPs-3 and SCAmpPs-4 genes were essentially identical (a single SCAmpPs PCR product– clone5- had a 1 bp deletion in this domain). The level of identity in the 5′ domains is consistent with the similar representation of SCAmpPs-3 and SCAmpPs-4 ESTs in the citrus phloem cDNA library (Additional file 3).

The level of sequence identity among the SCAmpPs-3 and SCAmpPs-4 genes will clearly prevent accurate assembly of individual members from short sequencing reads. The *C. sinensis* genome assembly [11] contains the intact gSCAmpPs-4-2 gene (Chr3 3.635) as well as two linked partial gSCAmpPs-3-1 genes (Chr3 3.555 Mb). The *C. clementina* assembly contains two SCAmpPs-4-related pseudogenes at 2.109 Mb (Additional file 6) and 2.118 Mb on Chr5, both interrupted by repetitive DNA sequences.

### SCAmpPs genomic architecture

**Figure 6:**
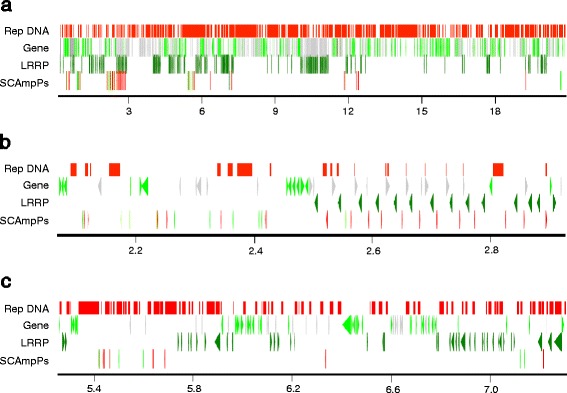
Distribution of SCAmpPs, Leucine-Rich Repeat Proteins, genes and repetitive DNA on *Citrus clementina* chromosome 5. **(a)** Positions 1–20.86 Mb of Chr5. SCAmpPs genes represented in the EST database are indicated in green, those not so represented in red. LRRP genes and pseudogenes are indicated in green. Other genes represented in the EST database are indicated in green, those not so represented are in grey. Ruler indicates position in Mb. **(b)** *C. clementina* Chr5 positions 2.07-2.92 Mb. **(c)** *C. clementina* Chr5 positions 5.25-7.30.

As shown in Figure 6a, 66 of the 102 SCAmpPs-related *C. clementina* genomic loci are located in the first 20.86 Mb of chromosome 5 [12]. Within this region, the distribution of SCAmpPs is non-random, most are present as members of localized SCAmpPs gene clusters [22]. This genomic region is also rich in arrays of Nucleotide Binding-Leucine Rich Repeat Protein (NB-LRRP) disease resistance gene arrays [23], several of which are proximal to the SCAmpPs gene assemblies. The clearest example of this is shown in Figure 6b. The region 2.50-2.89 Mb contains 13 tandemly repeated RPP13-like [24] NB-LRRP genes and 13 SCAmpPs genes. The duplications giving rise to this array resulted in duplication of a proximal SCAmpPs gene. Figure 6c showed the chromosome 5 region 5.27-5.96 Mb in which a SCAmpPs array is also flanked by duplicated NB-LRRP genes.

### Identification of SCAmpPs-4 Cyclic product by LC-MS

**Figure 7:**
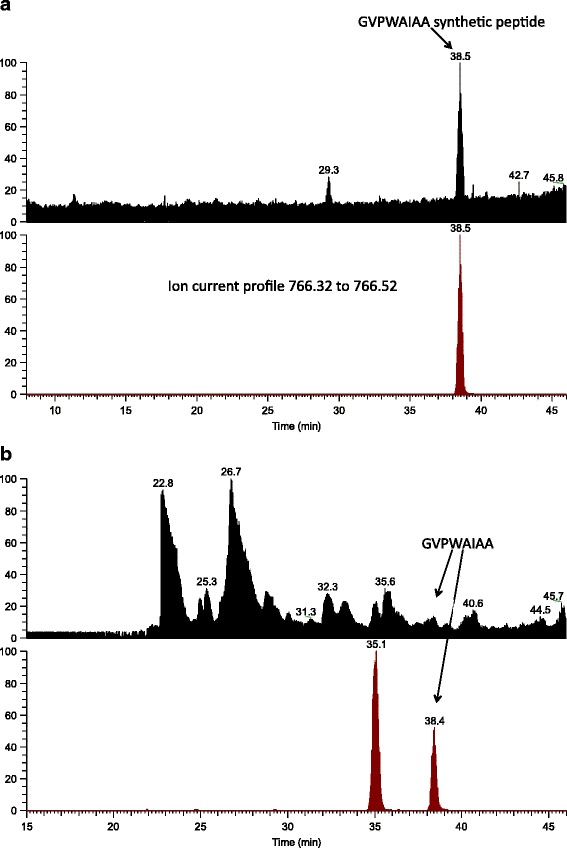
LC-MS of SCAmpPs cyclic peptides. **(a)**. HPLC-MS chromatogram of synthetic peptide GVPWAIAA. Upper trace is total ion current. Lower traced is the extracted ion current profile for the molecular ion (MH^+^) of cyclic GVPWAIAA. **(b)**. HPLC-MS chromatogram of the fractionated phloem extract. Upper trace is total ion current (TIC). Lower trace is ion current profile for 766.32-766.52 m/z consistent with the molecular ion (MH^+^) of GVPWAIAA. Peak at 38.4 minutes is consistent with retention time of synthetic cyclic peptide GVPWAIAA.

The SCAmpPs cyclic peptide products in Figure 2 are assigned based on precursor protein similarity to previously identified cyclic products (Figure 1) [8,9]. Mass spectral analysis of *C. clementina* phloem proteins was employed to verify this assignment. The molecular ion (MH^+^) of the cyclic SCAmpPs-4 peptide GVPWAIAA, has a calculated monoisotopic mass of 766.4252. The LC-MS profile of the phloem extract was compared to synthetic cyclic SCAmpPs-4 product in the expected molecular mass range (766.32-766.52 m/z) (Figure 7). The equivalence of the synthetic SCAmpPs-4 and the cyclic product from the phloem was established by consistent HPLC retention times and MS-MS HCD fragmentation of m/z 766.42 (Additional file 7).

The MS characterization of the SCAmpPs-4 chromatographic peak for both the synthetic peptide and the *C. clementina* phloem extract are shown in Additional file 8. The spectra averaged across the LC peak at the expected protonated cyclic peptide mass (766.42 m/z) also contains a separate, higher mass peak. The higher mass peak in the synthetic peptide sample (788.40 m/z) is consistent with the calculated molecular mass of the sodium adduct of the cyclic peptide (Additional file 8a). In contrast, the major peak in the phloem sample, measured as 804.38 m/z (Additional file 8b) is consistent with the calculated molecular mass of the potassium adduct of the cyclic peptide. Considering the LC conditions were the same in both cases, the relative intensity of the potassium adduct of the phloem sample compared to the synthetic sample indicates the source of the potassium, and therefore adduct, is the phloem. The relative difference in intensity is thought to be reflective of solution-phase binding [25]. MS-MS fragmentation of mass 804.38 m/z revealed minimal fragmentation at the HCD relative collision energy of 35 (data not shown) in contrast to significantly greater fragmentation observed for the non-adducted peptide, giving further evidence of stability of the molecule when complexed with potassium (Additional file 7).

To date, no biological role has been assigned to any of the orbitides [3]. Both the structure and cation binding properties of the SCAmpPs-4 (Additional file 7) open the possibility that this cyclic peptide represents a K^+^ ionophore [26]. This interpretation is also consistent with the fragmentation pattern of the K^+^ adduct, resulting from the conformational changes expected in the K^+^ complexed relative to the uncomplexed form of the cyclic peptide [27]. While alkali metal ion adducts are commonly observed in electrospray, the potassium adduct is observed only in the plant-derived sample (Additional file 8).

## Discussion

The biosynthesis of cyclic peptides occurs either through non-ribosomal pathways [28] or processing of ribosomally synthesized precursors [10]. Similar to *Citrus*, the Caryophyllaceae family members produce an array of 5–12 residue ribosomally synthesized small cyclic peptides derived from short precursor proteins [10]. Characterization of cyclic peptide biosynthesis in *Saponaria vaccaria* revealed the two-step processing of a precursor, involving an oligopeptidase cleavage of an amino terminal domain followed by an internal cyclization step resulting in removal of carboxy terminal residues [13]. With the exception of a conserved glycine residue at the amino terminal end of the cyclic peptide domain, the precursors for the *Saponaria vaccaria* peptides and citrus SCAmpPs share no sequence similarity. However, the precursor structures suggest a similar biosynthetic pathway in citrus [10].

Previously, SCAmpPs gene products have been proposed to be defense-related based on transcriptional profiles [7]. This interpretation is supported both by the structure of the final peptide product [29] and the chromosomal distribution of the SCAmpPs genes (Figure 6) [22]. The evolutionary profile of the SCAmpPs genes, in particular hypervariability of the predicted cyclic peptide domain, is also consistent with a defensive role [19,20,30].

The SCAmpPs composition, structure, expression, evolutionary profile and chromosomal distribution are consistent with designation as ribosomally synthesized antimicrobial peptides (AMPs) [1,31]. SCAmpPs predicted cyclic products are generally amphiphilic in nature and charged SCAmpPs are almost exclusively positive (Figure 2, Additional file 2), consistent with disruption of membrane permeability. The presence of the expected SCAmpPs-4 cyclic peptide in the phloem was confirmed by ESI-MS. In addition, these analyses suggested a non-covalent SCAmpPs-4-potassium complex. Relative binding efficiencies in non-covalent complexes, including polypeptide metal complexes, are known to be reflected in ESI-MS analyses [25]. The data is therefore consistent with strong binding of potassium by SCampPs-4 peptide in phloem, and opens the possibility that this peptide acts as a carrier-type ionophore capable of transporting K+ cations across a membrane [32].

The majority of the SCAmpPs loci identified on the *C. sinen*sis and *C. clementina* genome assemblies are not annotated. This is not unexpected given the small size of the precursor product, the interruption of this small domain by an intron, and the rapid divergence of genes for ribosomally synthesized peptide products [33].

While processing of the precursor to the cyclic product is unusual, the SCAmpPs-3 and SCAmpPs-4 genes also display an extraordinary evolutionary profile. The hypervariability of the 15–24 bp peptide domain (Figures 3 and 4) stands in contrast to the unexpected conservation of the 2.5 kb upstream from the peptide region (Figure 5). While the SCAmpPs-3 and SCAmpPs-4 genes encode radically different final cyclic products, these genes share >99.9% identity for the 2.5 kb 5′ to the peptide. This hyper-conservation may in fact be related to the unusual transcriptional profiles of the SCAmpPs-3 and SCAmpPs-4 genes. They are equally represented in the phloem cDNA library, each contributing >1% of the total transcripts. The maintenance of this level of transcription may preclude the expected accumulation of minor sequence changes (SNPs).

## Conclusion

We report here the description of the SCAmpPs gene family from citrus. The SCAmpPs predicted cyclic products have a number of properties that are consistent with designation as potential ribosomally synthesized antimicrobial peptides, including peptide structure, amino acid composition and potential cation binding properties. In addition, the expression patterns, evolutionary profile and chromosomal distribution of the genes encoding these peptides are consistent with this assignment in allowing evolutionary flexibility to respond to biotic challenges.

## Methods

### Identification of SCAmpPs genes

SCAmpPs genes were identified on the *Citrus clementina* (assembly v1.0) [12] and *Citrus sinensis* (assembly v1.0) [11] assemblies by Pustell matrix analysis [34] of individual down-loaded chromosomes (MacVector11.1) using the SCAmpPs precursor peptide consensus sequence (Figure 2). Individual SCAmpPs coding domains and flanking regions were then used in BLAST searches of the NCBI citrus EST database to identify related ESTs.

### PCR amplification of SCAmpPs-3 and SCAmpPs-4 genes

SCAmpPs genes were amplified from *Citrus* genomic DNA using synthetic oligonucleotide primers. SCAmpPs-3 and SCAmpPs-4 were amplified from *C. clementina* genomic DNA using oligos (For-WRB 2236 GTTACAGTTATGAACCCCTAACATTACTC and Rev-WRB 2239 ATAGAACATTTAAGATCGATGCTTAGC) based on the *C. clementina* genomic scaffolds. SCAmpPs-3 was also amplified from Carrizo citrange (*Citrus sinensis* ‘Washington’ sweet orange X *Poncirus trifoliata*) DNA using oligos (For-WRB 2228 CAGTTATGAACCCCTAACATTACTCATCC and Rev-WRB 2230 CTTTAGCACAAAGAGATCTCGATTCTC) based on the *C. sinensis* (For) and *Citrus* consensus (Rev) scaffold regions, respectively. PCR amplification was performed for 30 cycles using Phusion 2x High Fidelity Master Mix with HF buffer at manufacturer’s suggested annealing temperatures and conditions (New England Biolabs, Inc.-NEB). Amplified products were analyzed by 1% agarose gel electrophoresis (AGE), and PCR products were cloned directly into pCR Blunt II TOPO vector (Invitrogen™/Life Technologies) using the quick ligase protocol (NEB). Subsequent clones were identified via *EcoRI* restriction endonuclease digestion and AGE. SCAmpPs sequences were confirmed using fluorescent dideoxy sequencing of both strands employing overlapping synthetic oligonucleotide primers. Sequences were aligned, edited and compared (MacVector11.1). Complete sequences of the genomic PCR products are available on the USDA Public Citrus Genome Database (http://citrus.pw.usda.gov/).

### LC-MS-MS of the SCAmpPs-4 Synthetic Peptide and from Citrus clementina phloem

The SCAmpPs-4 s synthetic cyclic peptide GVPWAIAA was acquired from AnaSpec (Fremont, CA). Phloem tissue was prepared from the bark of nursery-grown *C. clementina* trees and powered under liquid nitrogen. A SCAmpPs cyclic peptide-enriched fraction was prepared by fractionating the methanol-soluble constituents from the phloem powder with ethyl acetate and ethanol and resuspended in water [35].

The synthetic peptide was solubilized with 0.1% formic acid in water (Optima LC/MS grade, Fisher Scientific, Waltham, MA), to approximately 1pM/uL. A portion of each sample was transferred to respective autosampler vials and placed in the autosampler of an EASY-nLC II interfaced to an Orbitrap Elite mass spectrometer (Thermo Scientific, San Jose, California) with a PicoChip nanospray source (New Objectives, Woburn, MA). For each LC-MS run, a 2uL portion of sample was loaded by the autosampler onto an EASY-column trap (2 cm, ID 100 μm, 5 μm, 120 Å, ReproSil-Pur C18-AQ) and washed with 16uL solvent A, then switched in-line with a 75 μm ID column containing 10 cm of 3 μm, 120 Å, ReproSil-Pur C18-AQ reverse phase packing (New Objectives, Woburn, MA). Samples were eluted into the Thermo Scientific Orbitrap Elite mass spectrometer with a binary gradient flow at a rate of 200 nL/minute. Solvent A was water, and Solvent B was acetonitrile, both solvents were Optima LC/MS grade containing 0.1% formic acid. The gradient was programed from 2% Solvent B to 16% Solvent B over 10 minutes, then to 36% solvent B at time 36 minutes, then to 80% solvent B at time 48 minutes, and held at 80% B for 8 minutes. Peptides were detected in the Orbitrap with the FT survey scan from 300 to 2000 m/z at a resolution of 30,000. The 6 most intense peaks above a threshold of 30,000 counts were subject to higher-energy C-trap dissociation (HCD) with normalized collision energy set to 35, default charge state set to 2, isolation width set to 2.0 m/z, and activation time set to 0.10 ms. Product spectra were recorded at 30,000 resolution with the low mass set to 50 Da. Dynamic exclusion was enabled for duration of 10 seconds with a repeat count of 1. Charge state screening allowed +1, +2, and +3 charge states to be selected for HCD fragmentation. Monoisotope precursor selection was enabled.

## Additional files

Additional file 1:
**Positions of SCAmpPs genes and pseudogenes on the C. clementina genome assembly.** Gene indicates representation (≥99%) in the NCBI EST DATA base. Additional file 2:
**Amino acid frequency of average citrus proteins compared to SCAmpPs peptides.** (a) Average amino acid frequency calculated using C. sinensis Chr7 proteins. Amino acid frequency is consistent with Jordan et al. (b) Amino acid frequency of combined SCAmpPs cyclic peptides from Figure 2. Additional file 3:
**Representation of SCAmpPs-2 (phloem-specific) and SCAmpPs-5 (abscission zone specific) in phloem and abscission zone EST libraries.** Complete citrus phloem (LIBEST_017673) and abscission zone (LIBEST_019157) EST libraries were downloaded from NCBI and sequences placed into contiguous DNA files. The EST libraries were probed by DNA matrix analysis (35 bp window, 85% identity) using SCAmpPs-2 (NCBI accession DR909920) and SCAmpPs-5 (NCBI accession FC872925). SCAmpPs-2, together with related SCAmpPs-3 and SCAmpPs-4 transcripts make up 5.5% of the transcripts in the phloem library. SCAmpPs-5, together with related SCAmpPs-6 transcripts make up 4.9% of the transcripts in the abscission zone library. Additional file 4:
**Alignment of related SCAmpPs ESTs.** (a) Alignment of SCAmpPs ESTs similar to the gene at position 7.120 Mb C. clementina Chr5, NCBI accession numbers indicated. (b) Alignment of SCAmpPs ESTs (NCBI accession numbers indicated). Coding domains, mismatches, position of the intronsindicated as in Figure 3. Additional file 5:
**Alignment of variable domains from SCAmpPs-3 and -4 promoters 130 bp 5′ to the transcription start.** Sequences of 4 SCAmpPs-3 and 3 SCAmpPs-4 gemomic PCR products aligned. Missmatch base pairs in red. Additional file 6:
**Structrue of SCAmpPs pseudogene at position 2.109 Mb C. clementina Chr5.** Exon, coding, cyclic peptide and variable regions are indicated as in Figure 7. The yellow boxes indicate positions of repetitive SINE and MITE elements. Additional file 7:
**MS-MS of SCAmpPs-4 synthetic peptide and phloem extract.** (a) MS-MS spectrum of m/z 766.42 collected during HPLC run of synthetic cyclic peptide GVPWAIAA. HCD collision energy 35 volts. (b) MS-MS spectrum of m/z 766.42 collected during HPLC run of methanol extract of phloem. HCD collision energy 35 volts. Additional file 8:
**Mass spectral analysis of SCAmpPs-4 chromatographic peaks from synthetic peptide and phloem extract.** (a) Synthetic peptide MS-1 mass spectrum chromatographic peak at 38.4 minutes. Peak at 766.4225 is consistent with calculated molecular weight of protonated cyclic peptide. Peak at 788.4038 is consistent with the calculated molecular weight of the sodium adduct of the cyclic peptide. Charge state (Z) and mass measurement error (given in parts per million) are indicated. (b) Methanol extract of phloem MS-1 mass spectrum chromatographic peak at 38.4 minutes. Peak at 766.4217 is consistent with calculated molecular weight of protonated cyclic peptide. Peak at 804.3769 is consistent with the calculated molecular weight of the potassium adduct of the cyclic peptide. Charge state (Z) and mass measurement error (given in parts per million) are indicated.

## Abbreviations

HLB: Huanglongbing
ESI-MS: Electrospray Ionization-Mass Spectrometry
HCD: Higher-energy C-trap dissociation
LC-MS: Liquid Chromatography- Mass Spectrometry
LC-MS-MS: Liquid Chromatography-Tandem Mass Spectrometry
NB-LRRP: Nucleotide Binding-Leucine Rich Repeat Protein
SCAmpPs: Small Cyclic Amphipathic Peptides
