# Supplementary material for: A family of small cyclic amphipathic peptides (SCAmpPs) genes in citrus
Source: BMC Genomics. 2015 Apr 16;16(1):303. doi: 10.1186/s12864-015-1486-4 (PMC4409773; doi:10.1186/s12864-015-1486-4)
Supplement: Additional file 1: — Positions of SCAmpPs genes and pseudogenes on the C. clementina genome assembly. Gene indicates representation (≥99%) in the NCBI EST DATA base. [file 12864_2015_1486_MOESM1_ESM.pdf]

Chr	Pos.	
4	0.509	Gene
4	0.530	Pseudogene
4	0.753	Pseudogene
4	0.755	Pseudogene
4	0.757	Pseudogene
4	7.214	Pseudogene
4	7.232	Pseudogene
4	7.247	Gene
4	7.254	Pseudogene
4	7.263	Pseudogene
5	0.441	Gene
5	0.459	Pseudogene
5	0.548	Pseudogene
5	0.553	Pseudogene
5	0.564	Pseudogene
5	0.899	Gene
5	0.904	Gene
5	0.911	Gene
5	0.923	Gene
5	0.935	Pseudogene
5	0.986	Gene
5	1.003	Gene
5	1.012	Pseudogene
5	2.109	Gene
5	2.111	Pseudogene
5	2.118	Pseudogene
5	2.174	Gene
5	2.189	Gene
5	2.235	Gene
5	2.236	Pseudogene
5	2.252	Pseudogene
5	2.265	Gene
5	2.325	Pseudogene
5	2.344	Pseudogene
5	2.364	Gene
5	2.408	Gene
5	2.412	Gene
5	2.420	Pseudogene
5	2.421	Pseudogene
5	2.523	Pseudogene
5	2.556	Gene
5	2.564	Pseudogene
5	2.595	Pseudogene
5	2.617	Pseudogene
5	2.651	Pseudogene
5	2.681	Pseudogene
5	2.710	Pseudogene
5	2.749	Pseudogene
5	2.774	Pseudogene
5	2.827	Pseudogene
5	2.858	Pseudogene
5	2.896	Pseudogene
5	5.422	Pseudogene

Chr	Pos.	
5	5.423	Gene
5	5.438	Pseudogene
5	5.442	Pseudogene
5	5.465	Pseudogene
5	5.466	Gene
5	5.501	Gene
5	5.600	Gene
5	5.640	Pseudogene
5	5.686	Pseudogene
5	6.335	Pseudogene
5	7.121	Gene
5	7.138	Gene
5	7.215	Pseudogene
5	11.833	Pseudogene
5	11.852	Pseudogene
5	11.855	Gene
5	12.314	Pseudogene
5	12.396	Pseudogene
5	19.085	Gene
5	19.249	Pseudogene
5	20.677	Gene
5	20.683	Gene
5	24.268	Pseudogene
5	24.270	Pseudogene
5	27.405	Gene
8	5.188	Gene
8	5.191	Pseudogene
8	20.458	Pseudogene
9	1.186	Gene
9	1.203	Pseudogene
9	1.226	Pseudogene
9	1.250	Gene
9	1.262	Pseudogene
9	12.311	Pseudogene
9	12.628	Pseudogene
9	12.648	Gene
9	12.684	Gene
9	21.347	Pseudogeneene
9	21.350	Pseudogeneene
9	21.354	Pseudogeneene
9	21.478	Pseudogeneene
9	21.486	Pseudogeneene
9	21.544	Pseudogeneene
9	21.548	Pseudogeneene
9	21.557	Two Pseudogenes
9	21.571	Pseudogene
9	21.586	Pseudogene
9	21.590	Pseudogene
9	21.592	Pseudogene
9	23.319	Gene
9	24.628	Pseudogenene

Additional File 1.
Positions of SCAmpPs genes and pseudogenes on the C. clementina genome assembly. Gene indicates representation (≥99%) in the NCBI EST DATA base.
