# Supplementary material for: A family of small cyclic amphipathic peptides (SCAmpPs) genes in citrus
Source: BMC Genomics. 2015 Apr 16;16(1):303. doi: 10.1186/s12864-015-1486-4 (PMC4409773; doi:10.1186/s12864-015-1486-4)
Supplement: Additional file 2: — Amino acid frequency of average citrus proteins compared to SCAmpPs peptides. (a) Average amino acid frequency calculated using C. sinensis Chr7 proteins. Amino acid frequency is consistent with Jordan et al. (b) Amino acid frequency of combined SCAmpPs cyclic peptides from Figure 2. [file 12864_2015_1486_MOESM2_ESM.pdf]

a Citrus Proteins

Aliphatic Index = 92.58

Non polar	Percent
A	6
V	7
L	11
I	6
P	4
M	2
F	4
W	1
**Polar**	**Percent**
G	6
S	8
T	5
C	2
Y	3
N	5
Q	4
**Acidic**	**Percent**
D	6
E	7
**Basic**	**Percent**
K	6
R	5
H	2

b Cyclic peptides

Aliphatic Index = 145.93

Non polar		No.	Percent
	A	9	3.9
	V	19	8.2
	L	42	18.2
	I	28	12.1
	P	25	10.8
	M	5	2.2
	F	27	11.7
	W	8	3.5
Polar:		No.	Percent
	G	27	11.7
	S	4	1.7
	T	9	3.9
	C	2	0.9
	Y	5	2.2
	N	7	3.0
	Q	4	1.7
Acidic:		No.	Percent
	D	0	0.0
	E	2	0.9
Basic:		No.	Percent
	K	1	0.4
	R	3	1.3
	H	4	1.7

Additional File 2.
Amino acid frequency of average citrus proteins compared to SCAmpPs peptides.
(a) Average amino acid frequency calculated using *C. sinensis* Chr7 proteins. Amino acid frequency is consistent with Jordan et al. (b) Amino acid frequency of combined SCAmpPs cyclic peptides from Figure 2.
