# Supplementary material for: A family of small cyclic amphipathic peptides (SCAmpPs) genes in citrus
Source: BMC Genomics. 2015 Apr 16;16(1):303. doi: 10.1186/s12864-015-1486-4 (PMC4409773; doi:10.1186/s12864-015-1486-4)
Supplement: Additional file 3: — Representation of SCAmpPs-2 (phloem-specific) and SCAmpPs-5 (abscission zone specific) in phloem and abscission zone EST libraries. Complete citrus phloem (LIBEST_017673) and abscission zone (LIBEST_019157) EST libraries were downloaded from NCBI and sequences placed into contiguous DNA files. The EST libraries were probed by DNA matrix analysis (35 bp window, 85% identity) using SCAmpPs-2 (NCBI accession DR909920) and SCAmpPs-5 (NCBI accession FC872925). SCAmpPs-2, together with related SCAmpPs-3 and SCAmpPs-4 transcripts make up 5.5% of the transcripts in the phloem library. SCAmpPs-5, together with related SCAmpPs-6 transcripts make up 4.9% of the transcripts in the abscission zone library. [file 12864_2015_1486_MOESM3_ESM.pdf]

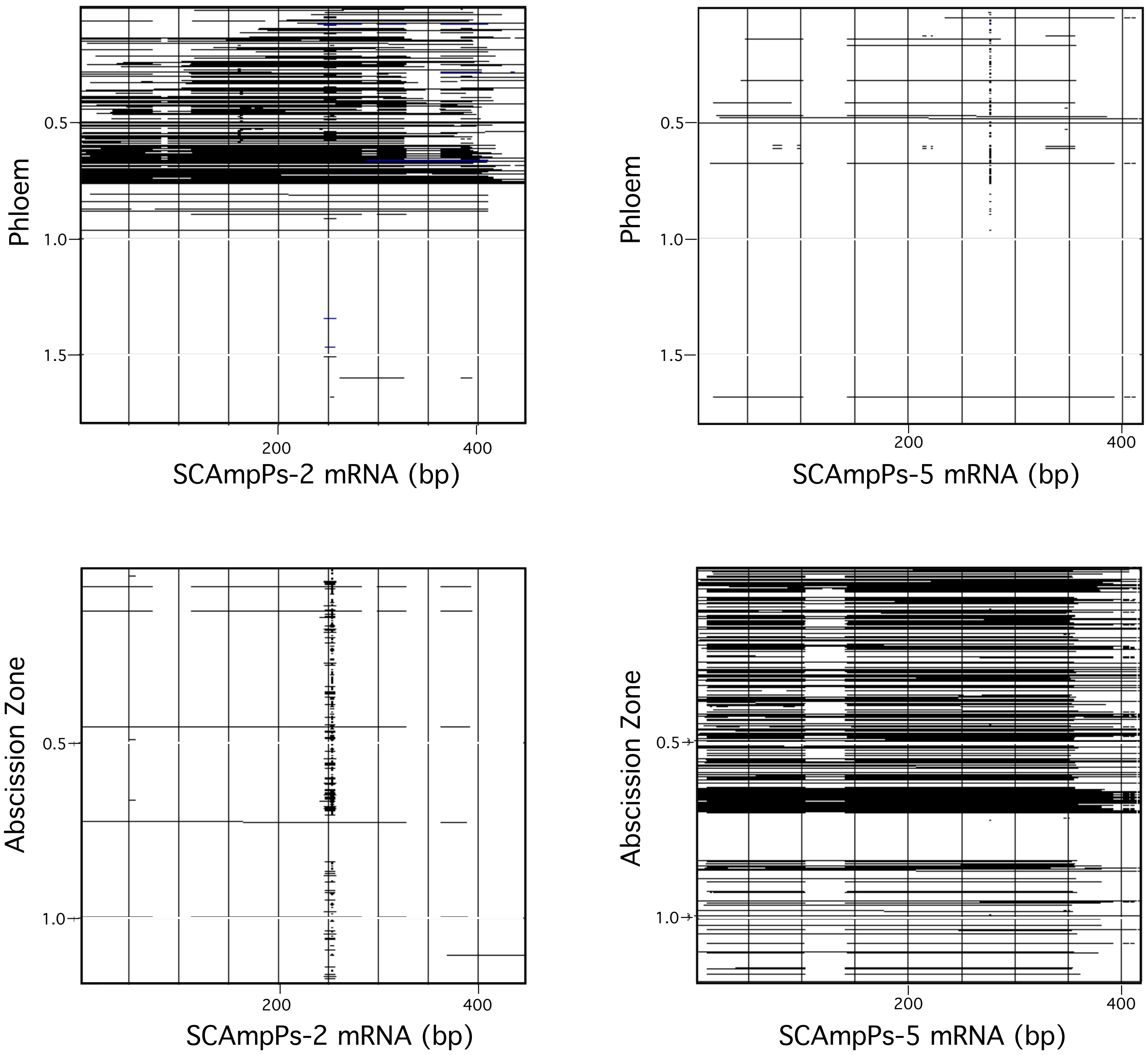

Additional File 3.

Representation of SCAmpPs-2 (phloem-specific) and SCAmpPs-5 (abscission zone specific) in phloem and abscission zone EST libraries. Complete citrus phloem (LIBEST_017673) and abscission zone (LIBEST_019157) EST libraries were downloaded from NCBI and sequences placed into contiguous DNA files. The EST libraries were probed by DNA matrix analysis (35 bp window, 85% identity) using SCAmpPs-2 (NCBI accession DR909920) and SCAmpPs-5 (NCBI accession FC872925).SCAmpPs-2, together with related SCAmpPs-3 and SCAmpPs-4 transcripts make up 5.5% of the transcripts in the phloem library. SCAmpPs-5, together with related SCAmpPs-6 transcripts make up 4.9% of the transcripts in the abscission zone library.
