# Supplementary material for: A family of small cyclic amphipathic peptides (SCAmpPs) genes in citrus
Source: BMC Genomics. 2015 Apr 16;16(1):303. doi: 10.1186/s12864-015-1486-4 (PMC4409773; doi:10.1186/s12864-015-1486-4)
Supplement: Additional file 4: — Alignment of related SCAmpPs ESTs. (a) Alignment of SCAmpPs ESTs similar to the gene at position 7.120 Mb C. clementina Chr5, NCBI accession numbers indicated. (b) Alignment of SCAmpPs ESTs (NCBI accession numbers indicated). Coding domains, mismatches, position of the intronsindicated as in Figure 3. [file 12864_2015_1486_MOESM4_ESM.pdf]

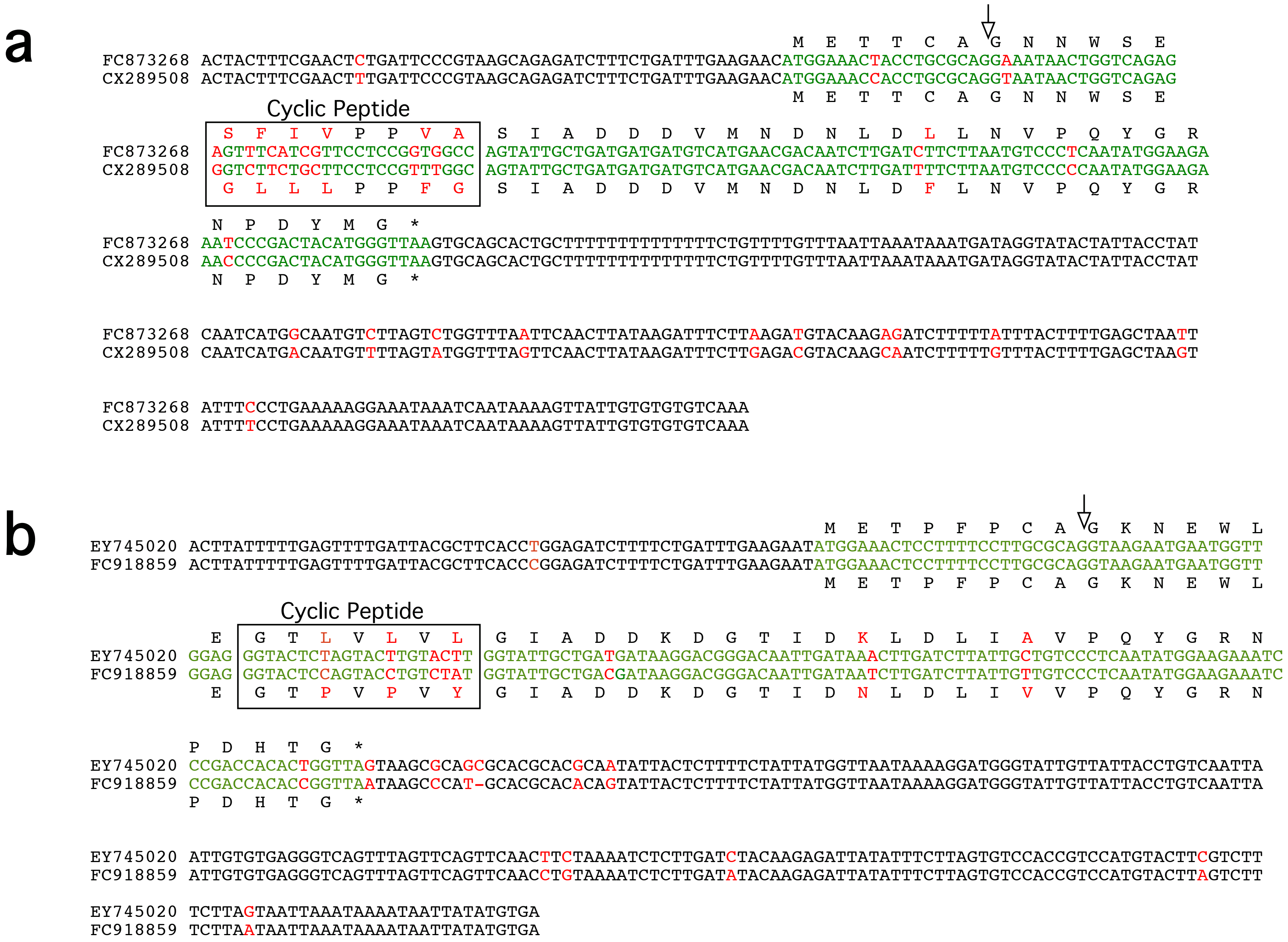

Additional File 4.

Alignment of related SCAmpPs ESTs. (a) Alignment of SCAmpPs ESTs similar to the gene at position 7.120 Mb C. clementina Chr5, NCBI accession numbers indicated. (b) Alignment of SCAmpPs ESTs (NCBI accession numbers indicated). Coding domains, mismatches, position of the intronsindicated as in Figure 3.
