# Supplementary material for: A family of small cyclic amphipathic peptides (SCAmpPs) genes in citrus
Source: BMC Genomics. 2015 Apr 16;16(1):303. doi: 10.1186/s12864-015-1486-4 (PMC4409773; doi:10.1186/s12864-015-1486-4)
Supplement: Additional file 5: — Alignment of variable domains from SCAmpPs-3 and -4 promoters 130 bp 5′ to the transcription start. Sequences of 4 SCAmpPs-3 and 3 SCAmpPs-4 gemomic PCR products aligned. Missmatch base pairs in red. [file 12864_2015_1486_MOESM5_ESM.pdf]

```
gSCAmpPs-4-1a  AAATATTTCAGAAAA-------------------GCGCGGTTCTGGCCACATTTTCCAAATTATTATTATTATTTTTTTGAAAGAAAAGAC
gSCAmpPs-4-1b  AAATATTTCAGAAAAATGAAAAACCACCGCGCTTTGGCGGTTCTGGCCACATTTTCCAAATTATTATTATTTTTTTTTTTAAAGAAAAGAC
```

```
gSCAmpPs-4-1a   AAATATTTCAGAAAAGCGCGGTTCTGGCCACATTTTCCAAATTATTATTATTATTTTTTTGAAAGAAAAGAC
gSCAmpPs-3-1    AAATATTTCAGAAAAGCGCGGTTCTGGCCACATTTTCCAAATTATTATTATTATTTTTTTGAAAGAAAAGAC
gSCAmpPs-3-1    AAATATTTCAGAAAAGCGCGGTTCTGGCCACATTTTCCAAATTATTATTATTATTTTTTTGAAAGAAAAGAC
gSCAmpPs-3-3    AAATATTTCAGAAAAGCGCGGTTCTGGCCACATTTTCCAAATTATTATTATTATTTTTTTGAAAGAAAAGAC

gSCAmpPs-4-1b  AAATATTTCAGAAAAATGAAAAACCACCGCGCTTTGGCGGTTCTGGCCACATTTTCCAAATTATTATTATTTTTTTTTTTAAAGAAAAGAC
gSCAmpPs-4-2   AAATATTTCAGAAAAATGAAAAACCACCGCGCTTTGGCGGTTCTGGCCACATTTTCCAAATTATTATTATTTTTTTTTTTAAAGAAAAGAC
gSCAmpPs-3-2   AAATATTTCAGAAAAATGAAAAACCACCGCGCTTTGGCGGTTCTGGCCACATTTTCCAAATTATTATTATTTTTTTTTTTAAAGAAAAGAC
```

Additional File 5.
Alignment of variable domains from SCAmpPs-3 and -4 promoters 130 bp 5' to the transcription start. Sequences of 4 SCAmpPs-3 and 3 SCAmpPs-4 gemomic PCR products aligned. Missmatch base pairs in red.
