# Supplementary material for: A family of small cyclic amphipathic peptides (SCAmpPs) genes in citrus
Source: BMC Genomics. 2015 Apr 16;16(1):303. doi: 10.1186/s12864-015-1486-4 (PMC4409773; doi:10.1186/s12864-015-1486-4)
Supplement: Additional file 6: — Structrue of SCAmpPs pseudogene at position 2.109 Mb C. clementina Chr5. Exon, coding, cyclic peptide and variable regions are indicated as in Figure 7. The yellow boxes indicate positions of repetitive SINE and MITE elements. [file 12864_2015_1486_MOESM6_ESM.pdf]

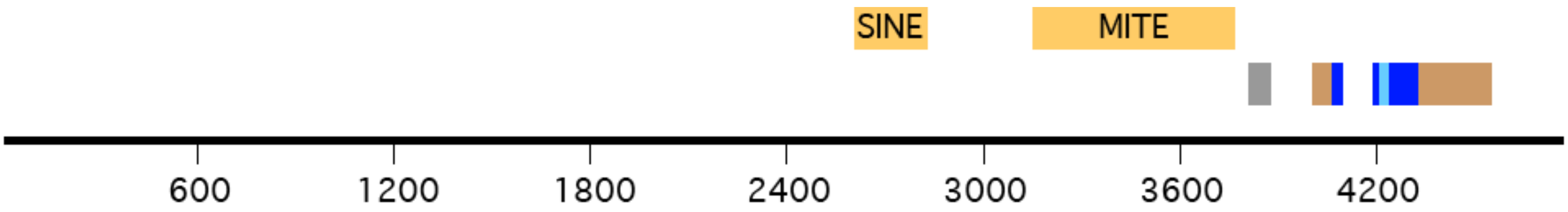

Additional File 6.

Structrue of SCAmpPs pseudogene at position 2.109 Mb *C. clementina* Chr5.

Exon, coding, cyclic peptide and variable regions are indicated as in Figure 5. The yellow boxes indicate positions of repetitive SINE and MITE elements.
