# Supplementary material for: A family of small cyclic amphipathic peptides (SCAmpPs) genes in citrus
Source: BMC Genomics. 2015 Apr 16;16(1):303. doi: 10.1186/s12864-015-1486-4 (PMC4409773; doi:10.1186/s12864-015-1486-4)
Supplement: Additional file 7: — MS-MS of SCAmpPs-4 synthetic peptide and phloem extract. (a) MS-MS spectrum of m/z 766.42 collected during HPLC run of synthetic cyclic peptide GVPWAIAA. HCD collision energy 35 volts. (b) MS-MS spectrum of m/z 766.42 collected during HPLC run of methanol extract of phloem. HCD collision energy 35 volts. [file 12864_2015_1486_MOESM7_ESM.pdf]

a

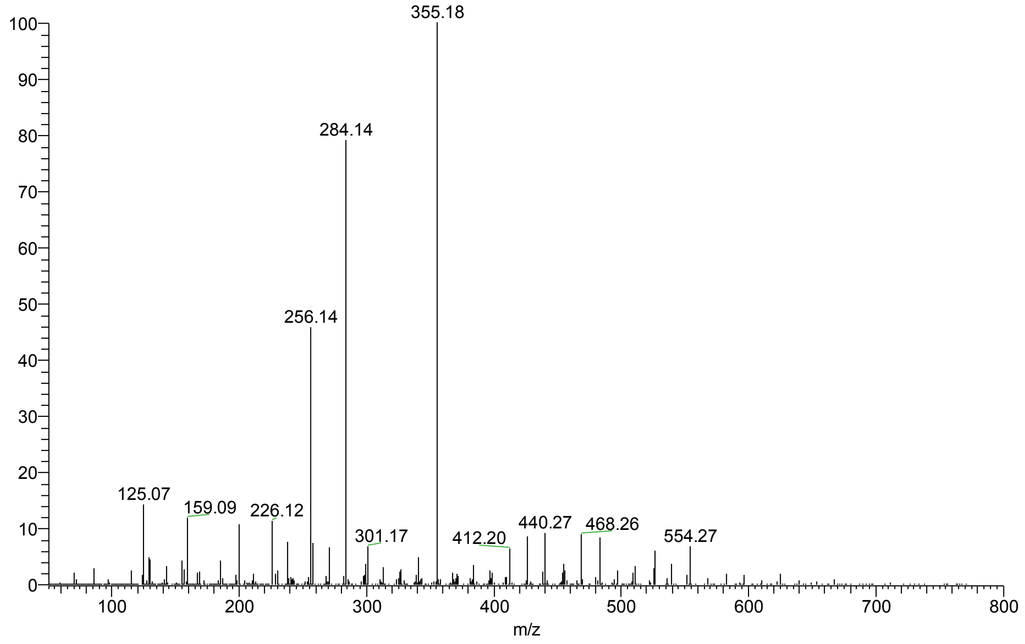

b

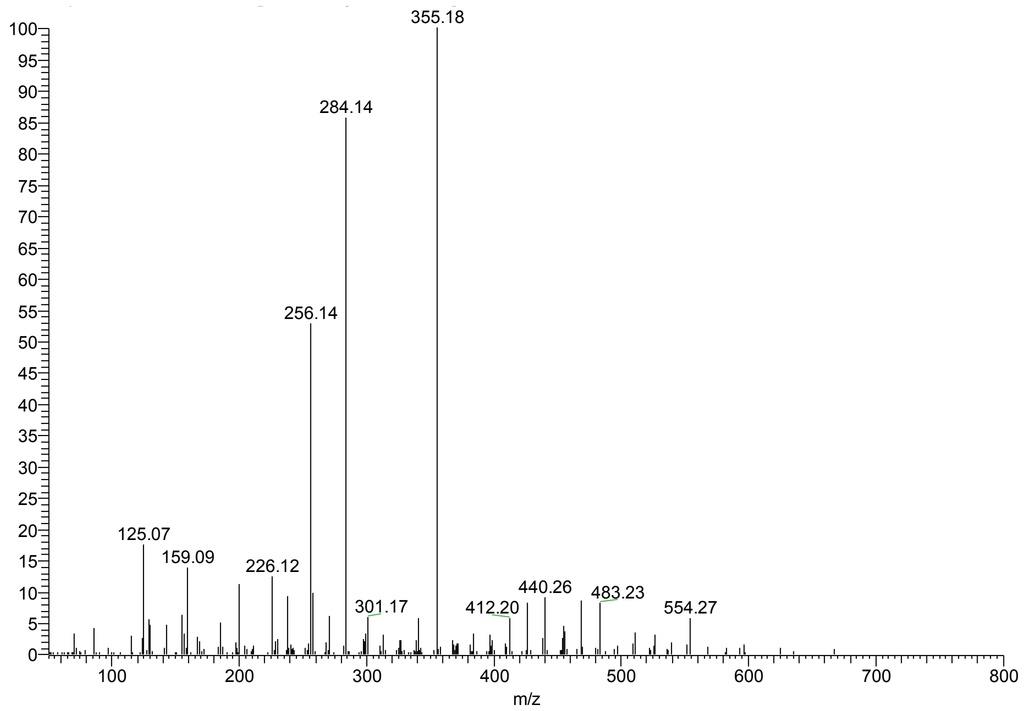

Additional File 7.
MS-MS of SCAmpPs-4 synthetic peptide and phloem extract. (a) MS-MS spectrum of m/z 766.42 collected during HPLC run of synthetic cyclic peptide GVPWAIAA. HCD collision energy 35 volts. (b) MS-MS spectrum of m/z 766.42 collected during HPLC run of methanol extract of phloem. HCD collision energy 35 volts
