# Supplementary material for: A family of small cyclic amphipathic peptides (SCAmpPs) genes in citrus
Source: BMC Genomics. 2015 Apr 16;16(1):303. doi: 10.1186/s12864-015-1486-4 (PMC4409773; doi:10.1186/s12864-015-1486-4)
Supplement: Additional file 8: — Mass spectral analysis of SCAmpPs-4 chromatographic peaks from synthetic peptide and phloem extract. (a) Synthetic peptide MS-1 mass spectrum chromatographic peak at 38.4 minutes. Peak at 766.4225 is consistent with calculated molecular weight of protonated cyclic peptide. Peak at 788.4038 is consistent with the calculated molecular weight of the sodium adduct of the cyclic peptide. Charge state (Z) and mass measurement error (given in parts per million) are indicated. (b) Methanol extract of phloem MS-1 mass spectrum chromatographic peak at 38.4 minutes. Peak at 766.4217 is consistent with calculated molecular weight of protonated cyclic peptide. Peak at 804.3769 is consistent with the calculated molecular weight of the potassium adduct of the cyclic peptide. Charge state (Z) and mass measurement error (given in parts per million) are indicated. [file 12864_2015_1486_MOESM8_ESM.pdf]

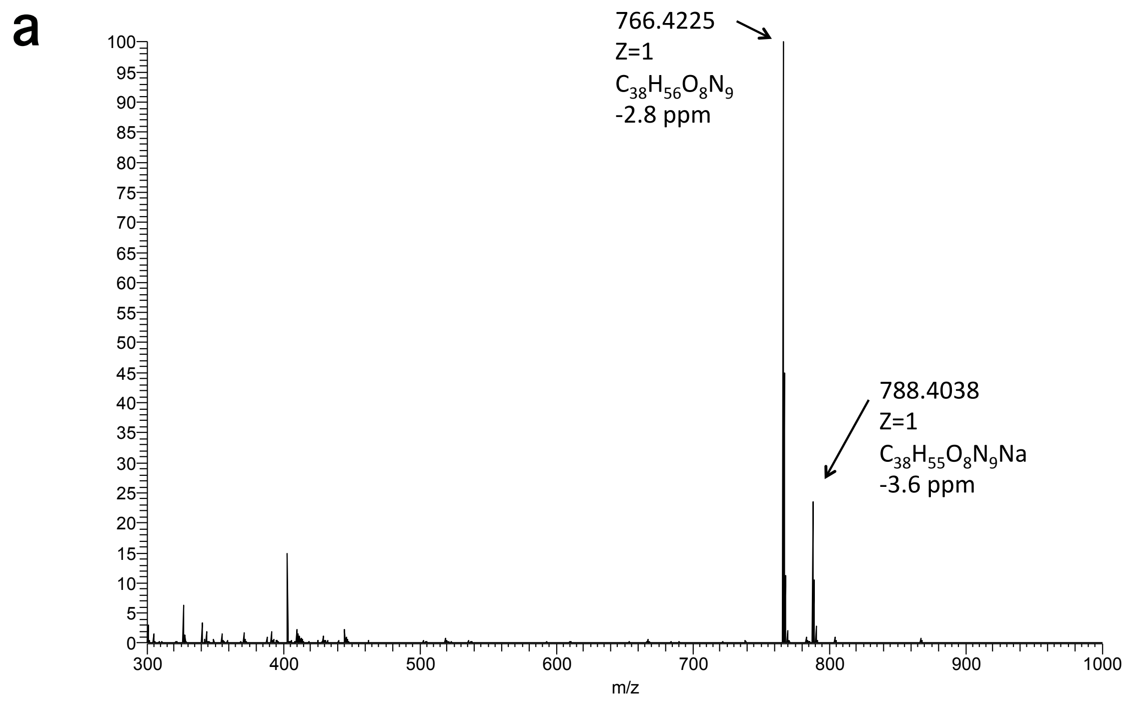

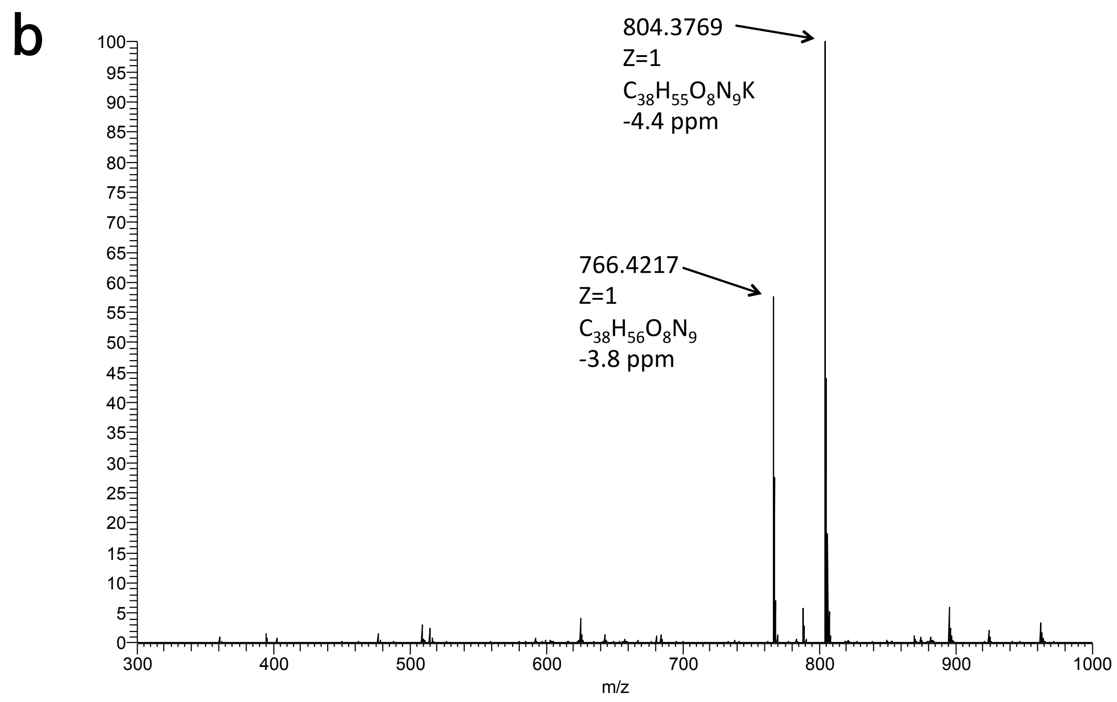

Additional File 8.

Mass spectral analysis of SCAmpPs-4 chromatographic peaks from synthetic peptide and phloem extract. (a) Synthetic peptide MS-1 mass spectrum chromatographic peak at 38.4 minutes. Peak at 766.4225 is consistent with calculated molecular weight of protonated cyclic peptide. Peak at 788.4038 is consistent with the calculated molecular weight of the sodium adduct of the cyclic peptide. Charge state (Z) and mass measurement error (given in parts per million) are indicated. (b) Methanol extract of phloem MS-1 mass spectrum chromatographic peak at 38.4 minutes. Peak at 766.4217 is consistent with calculated molecular weight of protonated cyclic peptide. Peak at 804.3769 is consistent with the calculated molecular weight of the potassium adduct of the cyclic peptide. Charge state (Z) and mass measurement error (given in parts per million) are indicated.
